# Experimental validation and molecular docking to explore the active components of cannabis in testicular function and sperm quality modulations in rats

**DOI:** 10.1186/s12906-022-03704-z

**Published:** 2022-08-26

**Authors:** Charles O. Nwonuma, Victoria C. Nwatu, Gomaa Mostafa-Hedeab, Oluyomi S. Adeyemi, Omokolade O. Alejolowo, Oluwafemi Adeleke Ojo, Sylvanus A. Adah, Oluwakemi J. Awakan, Charles E. Okolie, Nnaemeka Tobechukwu Asogwa, Udofia A. Inemesit, Godshelp O. Egharevba, Nada H. Aljarba, Saad Alkahtani, Gaber El-Saber Batiha

**Affiliations:** 1 Department of Biochemistry, College of Pure and Applied Sciences, Landmark University, Omuaran, Nigeria; 2 Pharmacology Department & Health Research, Unit-Medical College, Jouf University, Jouf, Saudi Arabia; 3 Pharmacology Department, Faculty of Medicine, Beni-Suef University, Beni Suef, Egypt; 4 Phytomedicine, Molecular Toxicology, and Computational Biochemistry Research Laboratory (PMTCB-RL), Department of Biochemistry, Bowen University, Iwo, Nigeria; 5 Department of Veterinary Physiology and Biochemistry, University of Ilorin, Ilorin, Nigeria; 6 Department of Microbiology College of Pure and Applied Sciences, Landmark University, Omu-aran, Nigeria; 7 Department of Medical Laboratory Sciences, Elizade University, Ondo State Ilara-Mokin, Nigeria; 8 Central Research Lab 123B, University Road Tanke, Ilorin, Kwara State Nigeria; 9 Department of Chemistry, University of Lagos, Lagos State, Nigeria; 10 Department of Physical Sciences, Landmark University, Omuaran, Kwara State Nigeria; 11 Department of Biology, College of Science, Princess Nourah bint Abdulrahman University, P.O. Box 84428, Riyadh, 11671 Saudi Arabia; 12 Department of Zoology, College of Science, King Saud University, P.O. Box 2455, Riyadh, 11451 Saudi Arabia; 13 Department of Pharmacology and Therapeutic, Faculty of Veterinary Medicine, Damanhour University, Damanhour, 22511 AlBeheira Egypt

**Keywords:** Cannabis, Antioxidants, Spermatozoa, Signal Transduction, Fertility, Phytochemicals

## Abstract

**Background:**

Data available support that ninety percent of male infertility cases are due to low sperm counts. There is a scarcity of data on the medicinal effects of cannabis on fertility. This study evaluated testicular function and sperm quality modulation with cannabis in rats.

**Methodology:**

Twenty-five male Wistar rats were randomly grouped into five: A, B, C, and D, each group have 5 rats. A (control): 0.2 ml 2% DMSO, B (vitamin C): 90 mg/kg body weight, C, D, and E were administered: 5 mg/kg, 10 mg/kg and 20 mg/kg body weight of ethanolic leaf extract of cannabis (ELEC) respectively. The rats were sacrificed 24 h after the last day of the 60 day oral administrations. Flavonoids were the predominant phytochemical present in the extract while quercetin, kemferol, silyman and gallic acid were identified.

**Results:**

The results showed a significant improvement (*p* < 0.05) in sperm quality and a significant increase in the concentrations of follicle-stimulating hormone, luteinizing hormone, triglycerides, cholesterol, and total protein determination compared to the normal control. Similarly, there was a significant increase (*p* < 0.05) in the activities of acid phosphatase, alkaline phosphatase, and superoxide dismutase compared to the normal control. RAC-alpha serine/threonine-protein kinase (AKT1)-silymarin complexes (-8.30 kcal/mol) and androgen receptor (AR)-quercetin complexes (9.20 kcal/mol) had the highest affinity.

**Conclusion:**

The antioxidant effects of the flavonoids in the ethanolic extract of cannabis may have protected testicular and sperm cells from oxidative damage. Biochemical processes and histopathological morphology were preserved by cannabis. The docking prediction suggests that the bioactive principle of cannabis may activate the androgenic receptors. The androgenic receptor modulation may be attributed to silymarin and quercetin.

**Supplementary Information:**

The online version contains supplementary material available at 10.1186/s12906-022-03704-z.

## Introduction

Cannabis has medicinal benefit other than reported use for pain relief [1, 2]. However, the psychoactive component, tetrahydrocannabinol (THC), listed as a schedule-1 illicit drug in the United Nations' Single Convention on Narcotic Drugs has overshadowed the medicinal use of cannabis which has prompted it none approval by governments to be used for therapeutic purposes. As a consequence of the government's destruction of cannabis confiscated from illicit users in Nigeria and other countries across the globe, considerable amounts of money are lost annually [3]. With over 15.3 billion dollars invested, Nigeria has one of the largest numbers of cannabis users in the world. Furthermore, research into the alternative use of this substance may help to redirect this fund into productive use instead of destroying the substance [4]. Likewise, recent studies have shown that cannabis can increase the quality of sperm and so can be used to treat infertility [5]. Sperm cells and testicular tissues are prone to oxidative degeneration by environmental toxicants, which was reported to be responsible for infertility in human. However, because of the antioxidant constituents of medicinal plants such as cannabis, they can serve as alternative therapy for infertility in humans or animals.

 Medicinal plants are robust repository of several antioxidants which makes them the alternative source for management of sexual dysfunction and other conditions associated with infertility [6]. Previous studies have shown that high phenolic and flavonoid compounds in plants are capable of scavenging free radicals, chelating metal-ion pro-oxidants and inhibiting some enzymes [7]. *Cannabis sativa L*., also known as hemp, is an annual herbaceous plant in the *cannabis* genus. The use of cannabis leaves for medicinal purpose dates back to the prehistoric times. It is thought to be one of the oldest medicinal herbs used by man. The testes are important male reproductive organs that are highly susceptible to toxic substances that can disrupt the blood-testis barrier, causing cell toxicity and, as a result, testicular dysfunction. The effect of these chemicals' inhibition can interfere with the synthesis and development of sperm cells, affecting the volume of sperm and its quality. Furthermore, estrogenic or anti-androgenic endocrine disrupting compounds may dominate the mechanism responsible for inhibiting the essential cellular process that controls the biosynthesis of testosterone in Leydig cells and androgen binding to the androgen receptor [8]. Because of the enormous medicinal value of cannabis leaf, with a focus on fertility enhancement, there is a need to scientifically validate the possible mode of action.

This research could lead to the discovery of a new drug that can be used instead of the current drug for the treatment of infertility. As a result, this study investigated the testicular function and sperm modulatory effect of ELEC in rats.

## Materials and methods

### Procurement and authentication of Cannabis

The dried cannabis leaves were obtained through an agreement with the National Drug Law Enforcement Agency (NDLEA) Ilorin Division. The leaf was authenticated at Ahmadu Bello University's Department of Botany in Zaria, Nigeria. It was given the voucher number ABU02438 and placed in the herbarium. All experimental protocols adhered to the ethical guidelines/regulations governing the use of plants.

### Preparation of cannabis extract

The ELEC was prepared by soaking 1400 g of pulverized cannabis leaf in 2.8 L 90% ethanol in a beaker. The mixture was stirred in an orbital shaker for 2 h and; it is then left to stand for 24 h. Subsequently, the mixture was filtered; the filtrate was concentrated at 60 °C with a rotary evaporator for 15 min. The semi-fluid extract was dried at 40 °C using a water bath to dry weight and the percentage yield was 7.7% [9].

### Quantitative phytochemical estimation

The phytochemical content of the plant sample was quantified spectrophotometrically by the respective method for each phytochemical form. The procedure by idumathi et al. [10] was used for the quantification of terpenoids. Furthermore, the quantities of alkaloids and glycosides were quantified by the methods described by Van-Burden and Robinson [11] and El-Olemy et al. [12] respectively. Flavonoids were described by the methods described by Bohm and Kocipai-Abyazan [13], while Kumar and Pandey [14] described the method for the quantification of Phenolics. Coumarins and steroids were quantified by the method described by Ejikeme et al. [15].

### High Performance Liquid Chromatography (HPLC–UV) analysis

The HPLC analysis of ELEC was performed using a chromatographic system (N 2000, Korea) that included an Autosampler (YL 9150) with a 100 μl fixed loop and a UV–Visible detector (YL9120). At room temperature, the separation was performed on an SGE Protocol PC18GP120 (250mm4.6 mm, 5 m) column. The mobile phase is methanol to water (70:30 v/v), and the separations were accomplished using isocratic mode, with elution at a flow rate of 1 ml/min. The samples were run for 15 min, and detection was achieved using a UV detector at 254 nm. The autochro -2000 software was used to record and process all chromatographic data. Gallic acid, silymarin, quercetin, and kaemferol were used as standard samples for the analysis.

### Animal procurement

Twenty-five male Wistar rats weighing 120–140 g were procured from the animal house of Department of Biochemistry, University of Ilorin. The rats were put in metal cages and were placed a well-ventilated space in the animal house of Landmark University and were allowed to acclimatize for seven (7) days before the experiment commenced. The animals were allowed access to their feed and water *ad libitium*.

### Experimental design

The experimental animals were randomly assigned to 5 treatment groups: A, control: 2% DMSO was administered, B: 90 mg/kg body weight vitamin C was administered. Similarly, groups C, D and E were administered 5, 10 and 20 mg/kg body weight of ELEC, respectively. The dose used in this present study was a modification to a previous study by Alagbonsi et al. [16]

### Animal euthanasia and collection of tissues

Following the experimental protocols for the handling of animals [17], the animals in this study were sacrificed 24 h after the last day of the 60-day experiment. The animals were euthanized after being anesthetized with 0.5 percent halothane and the jugular vein was then cut open to pump the blood into the sterilized sample container. Blood samples were further centrifuged at 5000 rpm for 5 min using a refrigerated centrifuge to separate serum. The serums collected were transferred to fresh sterile sample bottles and placed in the freezer before biochemical tests. The testes were taken from rats, homogenized, frozen and used for biochemical assays. A small portion of the tissue from the representative testes was cut and placed in 10% of the formal saline solution for histological examination.

### Preparation of organs for biochemical analysis

The testicular tissue was homogenized in an appropriate buffer. The homogenate was further centrifuged at 5000 × g for 10 min. The supernatant was removed, frozen and used for biochemical assays.

### Methods for the sperm analysis

The epididymis was harvested, quickly cleared of fatty tissue, and then weighed. The sperm per gram caudal epididymis was determined by weighing the caudal portion of the epididymis. 2010 WHO Manual was the reference used for the manual sperm examination [18]. Concentration was assessed using the Neubauer hemocytometer and the epididymal Sperm Concentration was counted by a modified method of Yokoi and Mayi [19]. Similarly, the Sperm Progressive Motility was evaluated by an earlier method by Sonmez et al. [20] Sperm count and sperm motility were evaluated at × 100 magnification under an Olympus light microscope equipped with a Makler counting chamber (Sefi-Medical Instruments, Haifa, Israel). Using an Olympus light microscope, two hundred sperm cells were examined at × 400 magnifications per animal to determine the morphological abnormalities. Olympus image analysis software was used for high-resolution 4-D acquisition through the light microscope.

### Assay of serum testosterone, luteinizing hormone (LH), and follicle- stimulating hormone (FSH) level

The LH FSH and testosterone were quantified in the serum on the procedure based on a solid phase enzyme-linked immunosorbent assay (ELISA).

### Biochemical assays

The method described by Kemp, A and Van Heijningen, AJ [21] was used to evaluate glycogen concentration, while the procedure described by Ilavarasan, R, Mallika, M [22] was followed for the determination of nitric oxide (NO) concentration. Rao and Ramakrishnan described the method used to evaluate 3-hydroxy-3-methylglutaryl-CoA reductase activity in the testis by measuring the ratio of 3-hydroxy-3-methylglutaryl-CoA (HMG-CoA) and mevalonate concentration [23]. Superoxide dismutase (SOD) activity in the testicular homogenate was estimated by the method described by Misra, HP and Fridovich, I [24]. Jollow, DJ, Mitchell, JR [25] described the method used to evaluate reduced glutathione (GSH) level in the testis. The assay method described by Aebi [26] was used to evaluate catalase activity. The lipid peroxidation was estimated by the method described by Kei [27]. The total protein concentration was estimated by the method described by Gornall, AG, Bardawill, CJ [28]. (ACP) and alkaline phosphatase (ALP) activities were evaluated by the method described by Wright, P, Leathwood, P [29]

### Tissue lipid extraction and estimation of concentration

Lipids in the freshly harvested testis were extracted following the method described by Folch, J, Lees, M [30]. The extracted mixture was further washed with 0.05 M KCl solution. Cholesterol and triglycerides were estimated in aliquots of chloroform–methanol extract. For the estimation of cholesterol concentration, 0.2 ml of the chloroform–methanol extract was evaporated to dryness at 60 °C. Furthermore, Triton X 100/chloroform mixture (1:1, v/v, 20 μl) was added to it to resolve the lipids and the solvent was evaporated again. 1 ml of cholesterol reagent from the kit (Spin React S.A., Santa Colona, Sant Esteve De Bas, Spain) was added to the evaporated extract, vortexed and left to incubate for 30 min at room temperature. Finally, the content of cholesterol was quantified spectrophotometrically [31]. Similarly, the concentration of triglycerides was determined in the chloroform–methanol extract according to the method described by Kriketos, AD, Furler, SM [32]. At 60 °C, an aliquot of the chloroform methanol extract was placed in Eppendorf tubes and allowed to evaporate to dryness. After allowing the dried extract to cool to room temperature, 97% of 200 ml of ethanol was added to the tube to re-suspend the triglyceride. 1 ml of the kit's triglycerides reagent (Spin React S.A., Santa Colona, Sant Esteve De Bas, Spain) was added to the evaporated extract, vortexed, and incubated at room temperature for 20 min. Triglyceride content was also measured spectrophotometrically. The plasma high-density lipoprotein (HDL-c) concentration was determined using the precipitation method, and the cholesterol concentration was estimated using a cholesterol kit.

### Histopathology

The testes were extracted and immediately fixed in 10% of the formal-saline solution for histopathological analysis. The tissue samples were embedded in paraffin and then cut into a cross-section of 4–5 mm thickness and stained with hematoxylin–eosin. Histological sections were analyzed and photographed by Olympus BX50 (Japan) photo-microscope. A Leica slide scanner was used to view the slides (SCN 4000, Leica Biosystems, Germany).

### Protein preparation

The molecular docking simulation study was carried out using the x-ray crystallographic structure of androgen receptor (AR) with the PDB code: 2Q7K and RAC-alpha serine/threonine-protein kinase (AKT1) with the PDB code: 3QKK. The crystal structures were curated by removing the bound ligands and water molecules as they are used to stabilize the protein structures with the help of AutoDock Tools version 1.5.6 [33]. Hydrogen atoms were added, Gasteiger charges added and saved for docking simulations.

### Ligand preparation

In this investigation, the reference ligands that were used are capivasertib and testosterone. The choice of capivasertib is its ability to inhibit all isoforms of AKT, while testosterone is known to mediate its action through AR. The geometries of gallic acid, kaempferol, capivasertib, quercetin and silymarin were built and preoptimized at the molecular mechanics level with UFF Force Field, using the Steepest Descent algorithm implemented in Avogadro version 1.2.0. Further optimizations of the obtained ground state geometries were performed at the B3LYP/6–311 + G(2d,p) level of theory in the phase using Gaussian 16 RevC.01 by means of resources provided by SEAGrid [34] facilities. The optimized structures were converted to.mol2 format and used for molecular docking simulation study.

### Molecular docking protocol

AutoDock Vina [35] was used for docking simulation. The docking parameters used for the 2Q7K-ligand simulation are: exhaustiveness = 20; center_x = 19.872083666, center_y = 7.17202171736 and center_z = 10.5685; size_x = 51.1804970921, size_y = 43.7164598852 and size_z = 53.5245303154. Similarly, the docking parameters used for the 3QKK-ligand simulation are: exhaustiveness = 20: center_x = 21.516, center_y = 2.3798 and center_z = 16.5016; size_x = 48.3612984443, size_y = 50.0442008591 and size_z = 59.6766584587. Throughout the docking experiment, the protein structures were kept rigid, while the torsions or degrees of freedom for the ligands were allowed full rotations. Nine conformational modes were obtained.

### Data analysis

The data were expressed as the mean of three ± SEM determinations; one-way variance analysis (ANOVA) followed by post hoc Tukey to compare mean and assess significant differences between variables at *p* < 0.05 between the variables. All statistical analyses were carried out using the Social Science Statistical Package, version 22 (SPSS Inc. Illinois, Chicago, USA).

## Results

The antioxidant phytochemical compounds, flavonoids, steroids, alkaloids, and phenolics were abundant in the ELEC (Table 1). The presence of gallic acid (0.0601 g/10 g), silymarin, (10.7060 g/10 g), quercetin (10.7060 g/10 g), kaemferol (10.7060 g/10 g), and unidentified compounds was revealed by HPLC analysis of flavonoids ( SF 1/ Table 1). When compared with the animals in the normal control group, there was a significant increase (*p* < 0.05) in sperm volume, concentration count, motile count, total sperm count, and percentage motility in the animals given vitamin C and ELEC doses (Table 2).

**Table 1:** Quantitative Phytochemical of ELEC and flavonoids compounds of HPLC

Phytochemicals	Quantity (mg/100 g)	Peak No	Peak ID	Ret Time	Height	Area	μg/10 g
Flavonoids	350.00 ± 0.17	1	Unidentified	0.107	5228.810	9588.600	0.0731
Steroids	292.96 ± 0.06	2	Gallic acid	0.840	1249.621	7881.600	0.0601
Alkaloids	138.32 ± 0.09	3	Unidentified	1.265	104.979	302.155	0.0023
Phenolic	113.80 ± 0.21	4	Silyman	1.407	1145.194	1789.270	10.7060
Coumarins	63.82 ± 0.01	5	Quercetin	2.715	274,110.781	5,833,277.50	44.4809
Terpenoids	13.92 ± 0.20	6	Unidentified	2.998	258,525.203	7,154,126.000	54.5528
		7	Kaemferol	5.615	3489.348	108,164.719	0.8248

*HPLC*: High Performance Liquid Chromatography

**Table 2:** Effect of ethanolic leaf extract of cannabis on sperm parameters in Wistar rats

Group	Normal control	Vitamin C	5 mg/kg BW	10 mg/kg BW	20 mg/kg BW
Sperm volume (mL)	1.59 ± 0.08^a^	1.71 ± 0.01^a^	1.58 ± 0.10^a^	1.72 ± 0.02^a^	1.69 ± 0.04^a^
Concentration count (× 10^6^ mL)	133.33 ± 6.67^a^	293.33 ± 6.67^b^	263.33 ± 13.33^ab^	183.33 ± 16.67^c^	216.67 ± 12.02^d^
Motile count (10^6^ mL)	56.67 ± 3.33^a^	246.67 ± 3.33^b^	276.67 ± 3.33^ab^	176.67 ± 3.33^c^	173.67 ± 6.67^c^
Total count (× 10^6^ mL})	193.33 ± 9.28^a^	504.33 ± 8.67^b^	442.00 ± 8.00^c^	317.00 ± 27.00^ab^	307.33 ± 30.67^ab^
Motility (%)	50.00 ± 0.00^a^	85.00 ± 1.00^b^	78.67 ± 12.86^a^	75.67 ± 14.33^b^	71.67 ± 14.33^b^
Fast	51.67 ± 3.33^a^	83.33 ± 0.88^a^	72.33 ± 9.59^a^	64.67 ± 12.99^a^	69.67 ± 13.45^a^
Slow	50.00 ± 4.53^c^	17.67 ± 2.88^a^	41.0000 ± 1.45^b^	51.0000 ± 1.00^c^	54.00 ± 2.08^c^
N	51.00 ± 2.08^a^	82.00 ± 2.00^c^	69.33 ± 11.39^a^	70.67 ± 12.99^a^	70.33 ± 13.86^a^
TD	22.67 ± 1.45^c^	8.00 ± 0.57^a^	11.67 ± 5.54^a^	13.33 ± 5.33^a^	13.00 ± 6.50^a^
ND	11.00 ± 0.57^c^	4.33 ± 0.33^a^	7.00 ± 3.05^a^	7.00 ± 3.00^a^	7.33 ± 3.84^a^
HD	13.00 ± 2.08^b^	5.00 ± 1.73^a^	6.67 ± 2.33^a^	6.00 ± 5.00^a^	7.33 ± 4.33^a^

The values are expressed as means of 3 replicates ± SEM

^abcd^*p* < *0.05* vs normal control

*N* Normal, *TD* Defected tail, *ND* Defected neck, *HD* Defected head

**Fig. 1:**
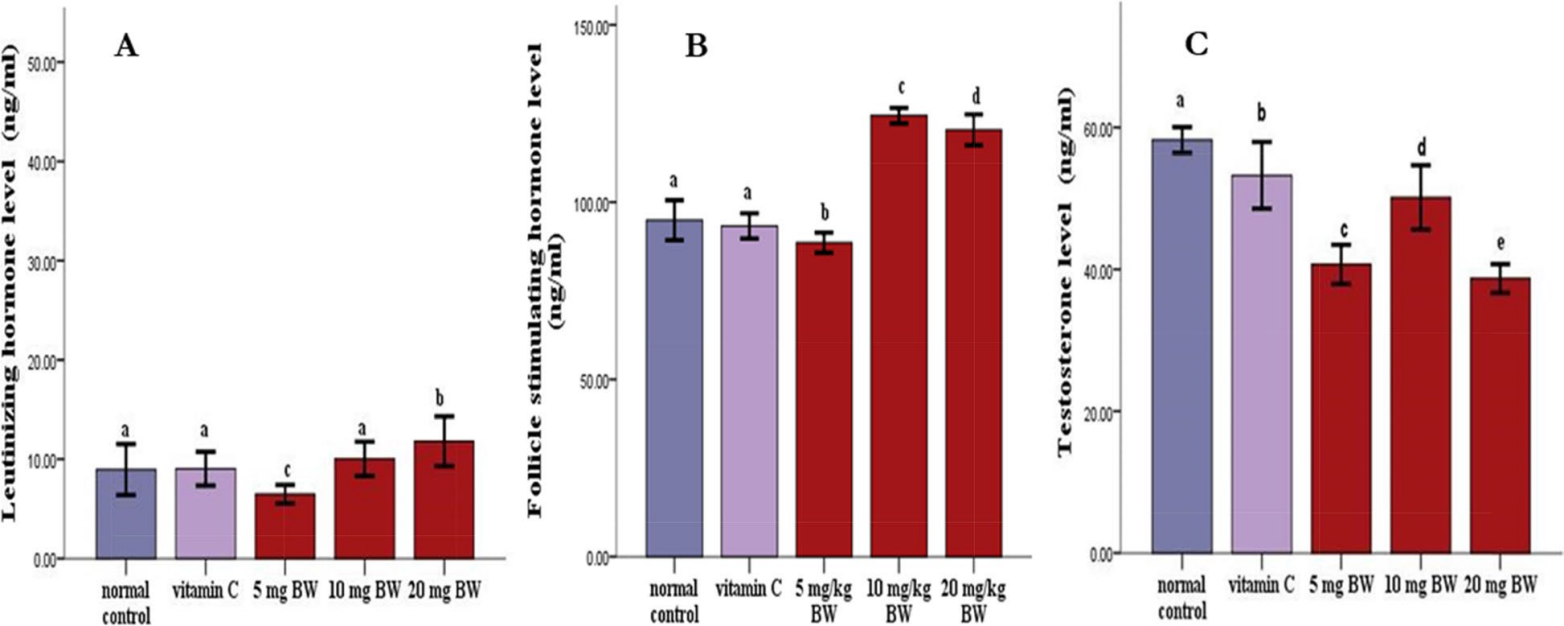
Serum levels of luteinizing hormone (**A)**, follicle stimulating hormone (**B)**, and testosterone (**C)** in rats treated with ELEC for 60 days. The values are expressed as means of three replicates ± SEM: ^abcd^ *p* < *0.05* vs normal control

**Fig. 2:**
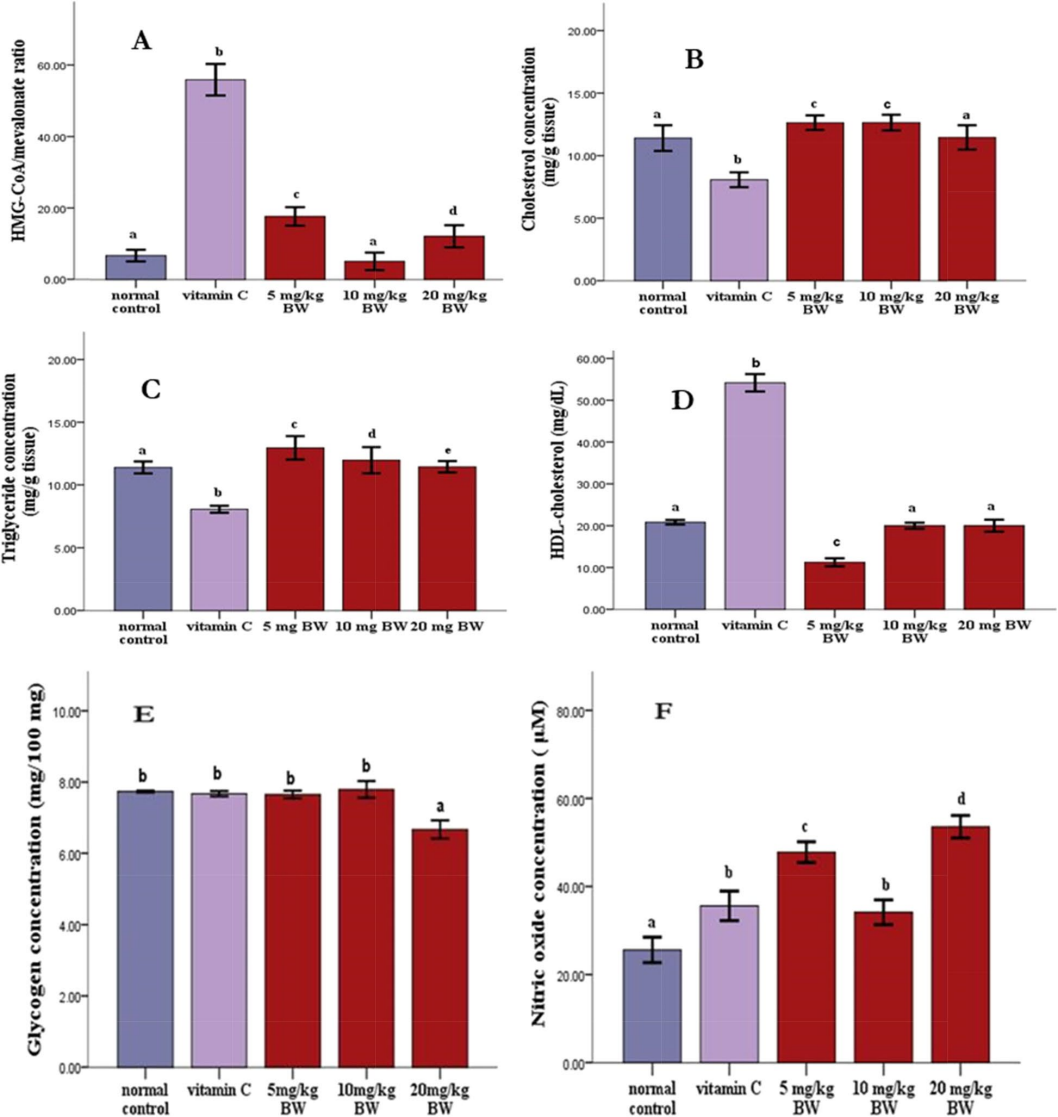
Testicular: HMG-CoA/Mevalonate ratio **A**, cholesterol concentration **B**, triglyceride concentration **C**, serum HDL concentration **D**, glycogen concentration (**E)** and nitric oxide concentration (**F)** in rats treated with ELEC for 60 days. The values are expressed as means of three replicates ± SEM: ^abcd^ *p* < *0.05* vs normal control

The number of fast-moving sperms was significantly higher (*p* < 0.05) in animals given vitamin C and the extract compared to normal control animals (Table 2). Similarly, animals given different doses of the extract and vitamin C had significantly (*p* < 0.05) more normal sperms than normal controls (Table 2). Rats in the treatment groups showed a greater percentage of weight change in the testis than did rats in the normal control group (Table 3). There was a significant increase (*p* < 0.05) in luteinizing hormone and follicle-stimulating hormone levels in the animals given the extract compared to the animals in the control and vitamin C groups (Fig. 1A and B). Furthermore, in comparison with the normal group, the rats administered the extract and vitamin C showed a significant decrease (*p* < 0.05) in testosterone levels (Fig. 1C). Furthermore, the HMG-CoA ratio decreased significantly (*p* < 0.05) in the animals administered the extract and normal control compared to the animals in the vitamin C group (Fig. 2A). Similarly, when compared with the vitamin C-treated group, the extract and normal control groups showed a significant increase (*p* < 0.05) in testicular cholesterol (Fig. 2B). Similarly, when compared to the normal control and vitamin C groups, there was a significant increase (*p* < 0.05) in testicular triglyceride levels in animals treated with the extract at lower doses (Fig. 2C). When compared with the animals in the vitamin C group, the serum HDL-c levels in the extract and normal control groups were significantly lower (*p* < 0.05) (Fig. 2D).

**Table 3:** The effect of ethanolic leaf extract of cannabis on the testicular weight change

Group	Initial weight	Final weight	% Weight change
Normal control	212.62 ± 13.62	218.14 ± 9.75	2.60 ↑
Vitamin C	211.00 ± 9.87	237.49 ± 11.18	12.60 ↑
5 mg/kg BW	199.67 ± 5.23	229.97 ± 7.29	15.16 ↑
10 mg/kg BW	198.33 ± 6.49	234.11 ± 15.74	18.04 ↑
20 mg/kg BW	208.33 ± 12.44	264.30 ± 16.08	26.90 ↑

The values are expressed as means of 3 replicates ± SEM

**Fig. 3:**
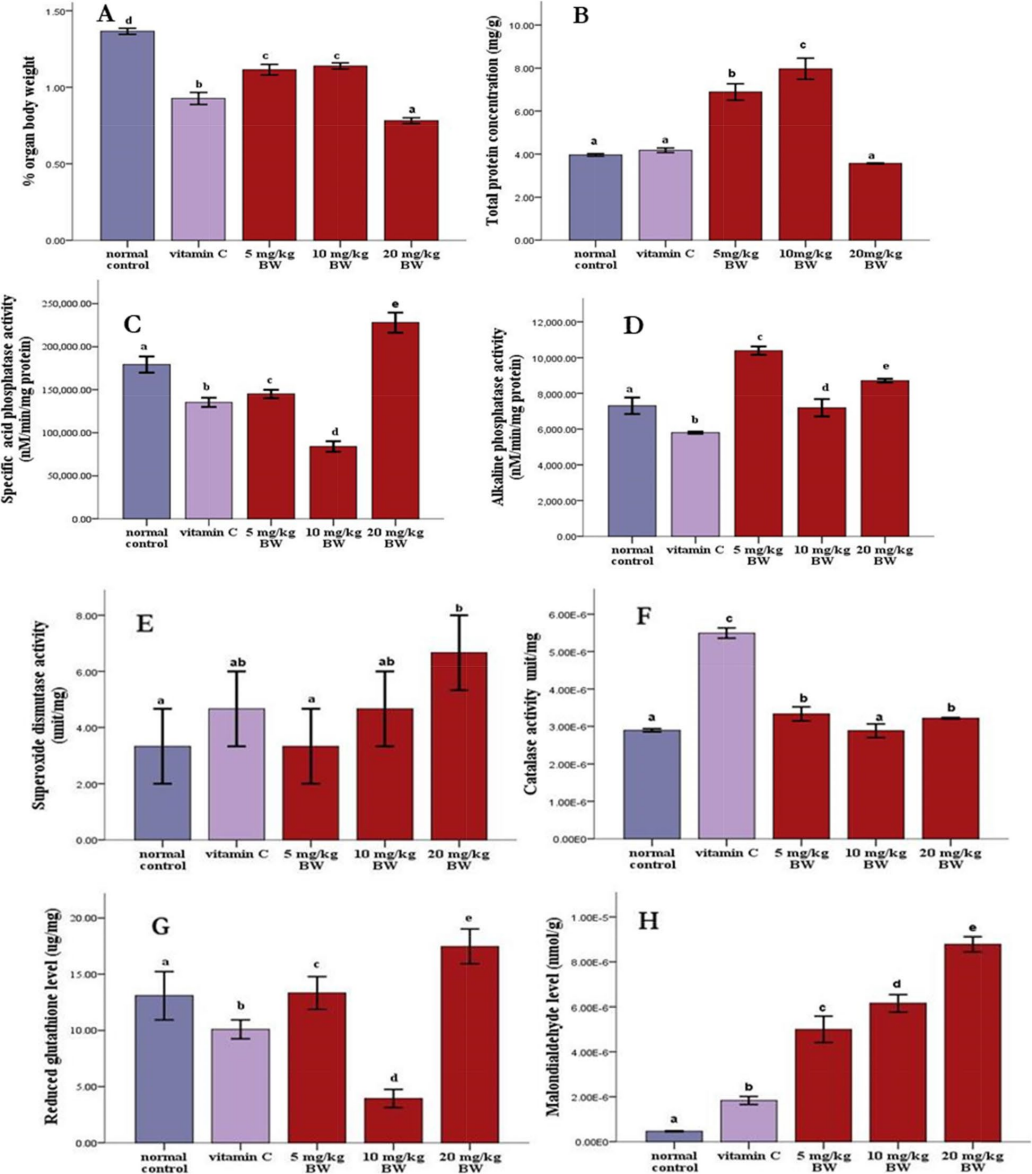
Testicular: organ body weights ratio **A**, total protein concentration **B**, specific acid phosphatase activity (**C**) and alkaline phosphatase activity (**D)**, superoxide dismutase activity (**E**), catalase activity (**F**), reduced glutathione level (**G**) and malondialdehyde level (**H**) in testis of rats treated with ELEC for 60 days. The values are expressed as means of three replicates ± SEM: ^abcd^ *p* < *0.05* vs normal control

The percentage organ-body ratio of the testes was significantly lower in rats treated with the extract and vitamin C compared to the normal control (Fig. 3A). In contrast, when compared with the normal control, the testicular total protein was significantly increased (*P* < 0.05) in the animals treated with the extract (Fig. 3B). Similarly, the activities of ACP and ALP were significantly increased (*p* < 0.05) in the extract groups compared to the animals in the control group (Fig. 3 C and D). When compared to the normal control, the extract and vitamin C had no effect on glycogen concentration in rats (Fig. 2E). However, when compared to animals in the normal control group, cannabis leaf extract and vitamin C significantly increased (*p* < 0.05) nitric oxide concentration in the rat (Fig. 2F). When the extract groups were compared to the control groups, there was a significant increase (*p* < 0.05) in SOD and catalase activity (Fig. 3 E and F).

**Fig. 4:**
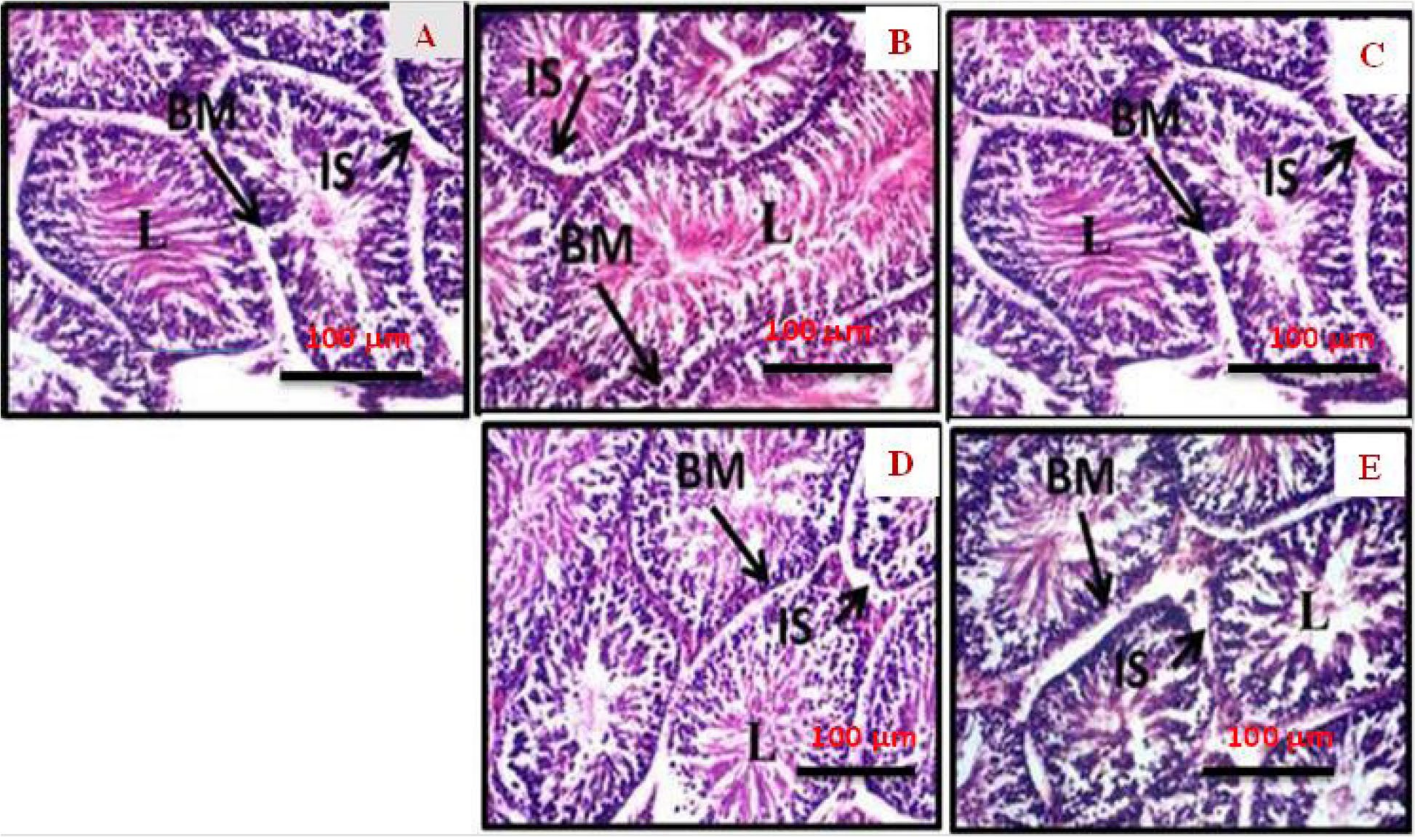
Photomicrograph (H & E X100 magnification) of the transverse section of testis of experimental anima l (**A**) normal control: showing degeneration of interstitial cells and increased intracellular spaces of the seminiferous tubules. **B** VIT C: showing normal histomorphology with typical seminiferous tubule containing different types of germ cells; spermatogonia lying on basement membrane with other cells proliferating in a centripetal direction. **C** 5 mg/kg body weight of ELEC; showing normal histomorphology with typical seminiferous tubule containing but with intercellular space. **D** 10 mg/kg body weight of ELEC; showing normal histomorphology with typical seminiferous tubule containing but with intercellular space. **E** 20 mg body weight of ELEC: abnormal widening of interstitial spaces (IS) with degeneration of interstitial cells. Increased intracellular spaces of the seminiferous tubules were also observed. BM: Basement membrane, IS: interstitial space, L, lumen

Similarly, there was a significant (*p* < 0.05) increase in GSH and malondialdehyde levels in the extract treated groups when compared to the normal control (Fig. 3 G and H). Furthermore, when compared to the normal control and vitamin C groups, the cell architecture of the testis of the animals treated with cannabis extract was normal, with no cell degeneration (Fig. 4 A-E). There are several types of interactions that determine ligand interactions with protein residues, among which are electrostatic interactions (hydrogen bonding), hydrophobic interactions, and halogen bonding.

**Fig. 5:**
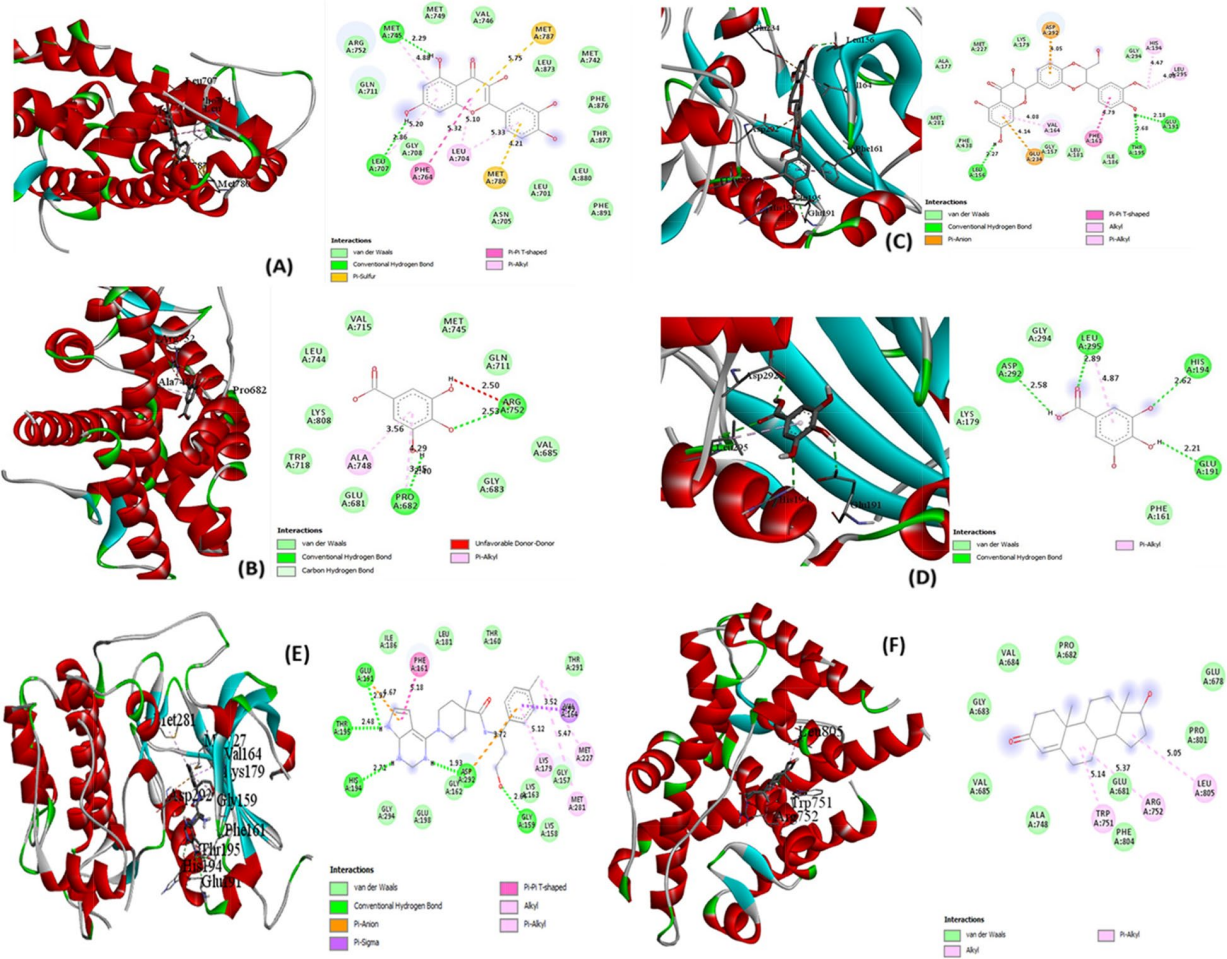
Interaction of (**A**) quercetin and (**B**) gallic acid with the protein residues of the androgen receptor (AR), and Interaction of (**C**) silymarin and (**D**) gallic acid with the protein residues of the RAC-alpha serine/threonine-protein kinase (AKT1), (**E**) capivasertib (reference compound) with the protein residues of the RAC-alpha serine/threonine-protein kinase (AKT1), and (**F**) testosterone (reference compound) interaction with the protein residues of the androgen receptor (AR)

AKT1 with silymarin and AR with quercetin have the best docking results, with binding energies of -9.40 and -9.20 kcal/mol, respectively, which are higher than capivasertib (-8.60 kcal/mol) and testosterone (-6.90 kcal/mol), the reference compounds (Table 4). The standard deviations are marginal, showing that each conformer does not greatly differ in their binding affinity. During the molecular docking experiment, the entire active space was sampled, and the pocket residues with constant binding interactions include LEU704, LEU707, and MET745 (Fig. 5A and B). The chemistry of interactions in AKT1-ligand complexes is dominated by conventional HB, electrostatic, and hydrophobic interactions, owing primarily to ASP292, GLU191, and THR195 (Fig. 5 C and D). The binding energy of AKT1- silymarin was -8.30 kcal/mol. and that of gallic acid was -5.90 kcal/mol, whereas the AR- quercetin complex binding energy was 9.20 kcal/mol. and that of AR- gallic acid was -5.60 kcal/mol. In general, ligands with AR have higher binding affinities than those with AKT1. Weak Van der Waals and hydrophobic interactions are the main interactions of testosterone, the reference compound for AR protein. TRP751, GLU681, and ARG752 were the pocket residues involved in these interactions but no hydrogen bonding interactions were observed. However, in addition to hydrophobic interactions, the AKT1 reference compound (capivasertib) is mediated by hydrogen bonding interactions.

**Table 4:** The binding energy, average binding energy of the nine conformational modes of each ligand

Protein	Ligand	Binding Energy (kcal/mol)	Average ΔG of ligand conformers (kcal/mol)
**AKT1**	Silymarin	-9.40	-9.00 $\pm$ 0.27
	Quercetin	-8.00	-7.56 $\pm$ 0.24
	Kaempferol	-7.60	-7.28 $\pm$ 0.16
	Gallic acid	-5.80	-5.47 $\pm$ 0.21
	Capivasertib	-8.60	-7.86 $\pm$ 0.32
**AR**	Quercetin	-9.20	-8.06 $\pm$ 0.69
	Kaempferol	-9.10	-7.94 $\pm$ 0.71
	Silymarin	-8.20	-7.58 $\pm$ 0.35
	Gallic acid	-5.60	-6.13 $\pm$ 0.27
	Testosterone	-6.90	-6.59 $\pm$ 0.19

*AKT1* RAC-alpha serine/threonine-protein kinase, *AR* Androgen receptor

## Discussion

Flavonoids have been widely reported to possess antioxidant potential which enables them to scavenge free radicals. The mechanisms of the antioxidant compounds are mainly by radical scavenging or metal ion chelation, and these activities are also based on configuration, substitution and the total number of hydroxyl groups. Flavonoids can also cause upregulation of antioxidant defense and can also inhibit reactive oxygen species (ROS) generation enzymes [36]. Furthermore, the optimal fertilizing capacity of the sperm cell will necessitate a controlled level of free radicals produced in the respiratory chain. As a result, a significant increase in the level of ROS would impede the upregulation of cyclic adenosine monophosphate (cAMP), which is responsible for the acrosome reaction; this would also result in lipid peroxidation of the sperm cell membrane and DNA fragmentation. Because of their antioxidant properties, vitamin C, Coenzyme Q, E, and glutathione are useful in the treatment of human infertility [37]. Previous studies have shown that the synergy of endogenous and exogenous antioxidant activity can reduce the effect of free radicals [38]. The increased flavonoid concentrations supported the antioxidant effect of this extract [39]. Similarly, previous research has shown that kaempferol and rutin provided antioxidant protection to sperm against ROS [40], which may be related to the study's improved sperm quality. This, in turn, resulted in improved sperm motility and viability. Sperm motility measures a sperm cell's ability to move properly across the vagina to the point where it can fertilize the ova, whereas insufficient sperm motility has been reported to be one of the most important parameters used to assess subfertility or infertility [41]. Furthermore, sperm viability and DNA fragmentation have been shown to contribute to fertility [42].

FSH represents the state of spermatogenesis as a result of feedback between the functions of the testis and the hypothalamus/pituitary glands. Spermatogenesis involves the interaction of hormones such as LH and FSH, which act on different cells of the testes due to the presence of their receptors: the FSHR is predominantly expressed in Sertoli cells, whereas the LHR is expressed in Leydig cells [43]. FSH acts on the Sertoli cell alone or in conjunction with testosterone to stimulate its proliferation and the production of signaling molecules and nutrients required for spermatid maturation [44]. As a result, the current study found increased levels of FSH and LH, indicating that cannabis extract may affect production. On the contrary, the extract appeared to reduce testosterone production in the Leydig cells, as evidenced by the lower serum testosterone levels reported in this study. The decrease in serum testosterone reported in this study, however, may not be below the threshold required for spermatogenesis. The high HMG-CoA/mevalonate ratio is an indicator of HMG-CoA reductase activity. The extract's increased reductase activity aided de novo synthesis of cholesterol, a precursor for testosterone and a building block for membranes in the testis. Furthermore, cholesterol is mobilized from HDL, although the extract did not alter the testicular HDL-c in this present study. One of the primary sources of cholesterol in testicular cells for the steroidogenic process is the cholesterol content of HDL-c [45]. Furthermore, triglyceride metabolism in the testes has been linked to male reproduction, potentially influencing spermatogenesis and sperm motility [46]. The increased triglyceride level in this study may have played a role in the improved sperm parameters.

The rise in MDA is a key indicator of lipid peroxidation. The dose-dependent increase in MDA could be due to the extract dosage used considering the long duration of this treatment, which could lead to accumulation in the tissue and thus predispose to oxidative stress. The ROS produced by this extract was superoxide anion, which explains the dose-dependent increase in SOD activity observed in this study. In the testes, SOD is a key antioxidant enzyme. It was reported to provide the first line of antioxidant enzyme protection against ROS. The inactivity of catalase reported in this study lends credence to the extract's antioxidant activity because the H_2_O_2_ produced by SOD did not increase the activity of CAT, implying that the H_2_O_2_ produced by SOD must have been scavenged by the extract's antioxidant function. The antioxidant effect of the extract could be the same reason for the higher level of GSH found in the study. The main source of energy to the testicular cells is glycogen. Glycogen found in Sertoli and spermatogonia cells nourishes the seminiferous tubule.

Glycogen content has also been reported to correlate with the levels of steroids [47]. In this study, the concentration of testicular glycogen was unaffected, indicating that the extract had no effect on glycogen synthesis in testicular cells. Meanwhile, nitric oxide synthase (NOS) activity has been reported in male reproductive organs [48]. Endothelial nitric oxide synthase is expressed in Sertoli and Leydig cells at various stages of spermatogenesis in testicular cells [49]. The increased nitric oxide activity may indicate that the extract increased the activity of the synthase. Furthermore, the reported decrease in testosterone levels in this study could be attributed to NO inhibition of testosterone synthesis in the Leydig cell [50]. The increased percentage weight change of the testis in this study could be due to the extract's protective effect against oxidative damage, which leads to cell death. The percentage of organ body weight is calculated by dividing the organ weight by the rat's weight and multiplying the result by 100. Because the percentage weight of the testes was normal, the decrease in the organ body weight ratio in this study was not associated with testicular cell death. However, this was due to the wide range of weights in the experimental animals. The testicular protein concentration refers to the total protein concentration in the testes, which includes enzymes involved in the physiological function of spermatogenesis and sperm cell differentiation [51]. The current study found an increase in total protein concentration, which could be an indication of the extract's effect. Furthermore, studies have shown that a change in ACP enzyme activity in matured testes along the spermatogenic cycle is also responsible for the maturation of spermatogonia to spermatozoa [52], and ACP activity has been reported to correlate with the number of germ cells present in the testis [53]. The increase in ACP activity reported in this study indicated an intact testicular cell that will later differentiate into normal and healthy sperm cells. The increase in ALP activity in this study also supported the extract's spermatogenic function. Sadik described ALP's spermatogenic function, which involves nuclear protein, nucleic acid, and phospholipid biosynthesis, phosphate ester cleavage, and mobilization of spermatogenic carbohydrates and lipid metabolites [50].

Because the Sertoli cell and spermatogonia cells host spermatogenesis, disruption of the cytoskeleton or degeneration of these testicular cells may affect sperm production [54]. The normal cell architecture of the testes reported in the current study lends credence to the antioxidant property of the extract or to the activation of a signaling pathway [55]. The signaling pathway caused by the interaction of silymarin and AKT1 could be the mechanism responsible for the improved sperm quality by cannabis extract. Signaling pathways are required for spermatogenesis to maintain a precise balance of cell survival, proliferation, and differentiation [56]. Furthermore, the increase in testicular weight is a function of germ and Sertoli cell proliferation and survival. In normal physiological conditions, AKT1 signaling regulates germ cell elimination via apoptosis, which may increase after testicular injury [57]. AR has been found in the nuclei of Sertoli, Leydig, and peritubular myoid cells in the testis, as well as epididymal epithelial and stromal cells [58]. Androgens are steroid hormones that are required for normal male reproductive development and function. Androgens act via AR, and its signaling in the testis is required for spermatogenesis [59]. The high binding energies of androgen receptor-quercetin or kempferol complexes could be a predictor of the mechanism for improved testicular function and sperm quality, according to the study, which also backed up a previous study that found kaempferol could improve mouse Leydig and Sertoli cell viability, male reproductive organ weights, and sperm quality [60].

## Conclusion

Ethanolic leaf extract of cannabis has antioxidant properties that may have protected testicular cells and sperm cells from oxidative damage due to its high flavonoid content. In the same way, cannabis improved testicular biochemical processes and histopathological cell morphology. The activation of signal pathways in androgenic cells could be responsible for the improved sperm quality. The results of this study's docking indicate that cannabis' bioactive principle (s) may activate androgenic receptors, triggering a cascade mechanism required for testicular function and spermatogenesis. Silymarin and quercetin may be responsible for the androgenic receptor modulation.

## Supplementary Information

**Additional file 1.** HPLC chromatogram of ethanolic leaf extract of cannabis.

## Data Availability

The data sets used and/or analyzed during the current study are available from the corresponding author on reasonable request.

## Abbreviations

ELEC: Ethanolic leaf extract of cannabis
cAMP: Cyclic adenosine monophosphate
AKT1: RAC-alpha serine/threonine-protein kinase
AR: Androgen receptor
HPLC: High Performance Liquid Chromatography
SF: Supplementary file
