# Correction: Experimental validation and molecular docking to explore the active components of cannabis in testicular function and sperm quality modulations in rats

**DOI:** 10.1186/s12906-022-03731-w

**Published:** 2022-10-07

**Authors:** Charles O. Nwonuma, Victoria C. Nwatu, Gomaa Mostafa‑Hedeab, Oluyomi S. Adeyemi, Omokolade O. Alejolowo, Oluwafemi Adeleke Ojo, Sylvanus A. Adah, Oluwakemi J. Awakan, Charles E. Okolie, Nnaemeka Tobechukwu Asogwa, Inemesit A. Udofia, Godshelp O. Egharevba, Nada H. Aljarba, Saad Alkahtani, Gaber El‑Saber Batiha

**Affiliations:** 1 Department of Biochemistry, College of Pure and Applied Sciences, Landmark University, Omuaran, Nigeria; 2 Pharmacology Department & Health Research, Unit-Medical College, Jouf University, Jouf, Saudi Arabia; 3 Pharmacology Department, Faculty of Medicine, Beni-Suef University, Beni Suef, Egypt; 4 Phytomedicine, Molecular Toxicology, and Computational Biochemistry Research Laboratory (PMTCB‑RL), Department of Biochemistry, Bowen University, Iwo, Nigeria; 5 Department of Veterinary Physiology and Biochemistry, University of Ilorin, Ilorin, Nigeria; 6 Department of Microbiology College of Pure and Applied Sciences, Landmark University, Omu‑aran, Nigeria; 7 Department of Medical Laboratory Sciences, Elizade University, Ondo State, Ilara‑Mokin, Nigeria; 8 Central Research Lab 123B, University Road Tanke, Ilorin, Kwara State Nigeria; 9 Department of Chemistry, University of Lagos, Lagos State, Nigeria; 10 Department of Physical Sciences, Landmark University, Omuaran, Kwara State Nigeria; 11 Department of Biology, College of Science, Princess Nourah bint Abdulrahman University, P.O. Box 84428, 11671 Riyadh, Saudi Arabia; 12 Department of Zoology, College of Science, King Saud University, P.O. Box 2455, 11451 Riyadh, Saudi Arabia; 13 Department of Pharmacology and Therapeutic, Faculty of Veterinary Medicine, Damanhour University, 22511 Damanhour, AlBeheira, Egypt

**Correction: BMC Complement Med Ther 22, 227 (2022)**.

10.1186/s12906-022-03704-z.

Following publication of the original article [1], the authors identified an error in the author name Inemesit A. Udofia, and Funding section statement.

The incorrect author name is: Udofia A. Inemesit.

The correct author name is: Inemesit A. Udofia.

The author group has been updated above and the original article [1] has been corrected.
