# Supplementary material for: Experimental validation and molecular docking to explore the active components of cannabis in testicular function and sperm quality modulations in rats
Source: BMC Complement Med Ther. 2022 Aug 26;22:227. doi: 10.1186/s12906-022-03704-z (PMC9414411; doi:10.1186/s12906-022-03704-z)
Supplement: Supplementary file 1 — Additional file 1. HPLC chromatogram of ethanolic leaf extract of cannabis. [file 12906_2022_3704_MOESM1_ESM.docx]

**Supplementary file**
